# Integrating Radiologic and Clinical Features to Predict VSX1 Expression in Clear Cell Renal Cell Carcinoma

**DOI:** 10.3390/curroncol32060348

**Published:** 2025-06-12

**Authors:** Federico Greco, Andrea Panunzio, Daniele Sergi, Marco Cataldo, Caterina Bernetti, Alessandro Tafuri, Bruno Beomonte Zobel, Carlo Augusto Mallio

**Affiliations:** 1 Department of Radiology, Cittadella della Salute, Azienda Sanitaria Locale di Lecce, Piazza Filippo Bottazzi, 2, 73100 Lecce, Italy; 2 Research Unit of Radiology, Department of Medicine and Surgery, Università Campus Bio-Medico di Roma, Via Alvaro del Portillo, 21, 00128 Roma, Italy; c.bernetti@policlinicocampus.it (C.B.); b.zobel@policlinicocampus.it (B.B.Z.); c.mallio@policlinicocampus.it (C.A.M.); 3 Department of Urology, “Vito Fazzi” Hospital, Piazza Filippo Muratore, 1, 73100 Lecce, Italy; andrea.panunzio@asl.lecce.it (A.P.); alessandro.tafuri@asl.lecce.it (A.T.); 4 Department of Diagnostics, “Vito Fazzi” Hospital, Azienda Sanitaria Locale di Lecce, Piazza Filippo Muratore, 1, 73100 Lecce, Italy; daniele.sergi@asl.lecce.it; 5 Apphia srl, Via per Monteroni, 73100 Lecce, Italy; marco.cataldo@apphia.it; 6 Fondazione Policlinico Universitario Campus Bio-Medico, Via Alvaro del Portillo, 200, 00128 Roma, Italy

**Keywords:** clear cell renal cell carcinoma, computed tomography, radiogenomics, VSX1

## Abstract

**Background:** Visual system homeobox 1 (VSX1) is a transcription factor recently implicated in promoting tumor aggressiveness in clear cell renal cell carcinoma (ccRCC). While its molecular role is emerging, the radiogenomic characteristics associated with VSX1 expression remain unexplored. This study aims to investigate the computed tomography (CT) imaging features linked to VSX1 expression, hypothesizing a correlation with radiologic markers of aggressive tumor behavior. **Methods:** A retrospective analysis was conducted using imaging and clinical data from 194 ccRCC patients obtained from The Cancer Genome Atlas and The Cancer Imaging Archive. CT scans were evaluated for multiple tumor characteristics, including necrosis, collecting system invasion, and perinephric fat stranding. Patients were stratified into VSX1-positive and -negative groups. A Lasso logistic regression model was applied to identify the most predictive imaging and clinical variables associated with VSX1 expression. A predictive score and probability nomogram were developed and evaluated. **Results:** VSX1 expression was positive in 19.1% of patients. Key features significantly associated with VSX1 positivity included male sex, tumor necrosis > 66%, collecting system invasion, and perinephric fat stranding. The Lasso model demonstrated good discriminatory power (area under the curve 0.75), and the predictive score effectively stratified patients based on estimated expression risk. **Conclusions:** VSX1-positive ccRCC presents with a distinct radiogenomic phenotype marked by imaging features of known aggressiveness. The integration of radiologic and genomic data allows for non-invasive risk stratification and may inform future personalized therapeutic strategies. External validation is needed to confirm clinical applicability.

## Simple Summary

Clear cell renal cell carcinoma is the most common type of kidney cancer and can show aggressive behavior. Recent research suggests that the gene VSX1 is associated with worse outcomes in these tumors. In this study, we analyzed CT scans and clinical data from 194 patients to investigate whether specific imaging features are linked to VSX1 expression. We found that VSX1-positive tumors were more common in male patients and were associated with tumor necrosis greater than 66%, collecting system invasion, and perinephric adipose tissue stranding. Based on these features, we created a predictive score to estimate the likelihood of VSX1 expression. This non-invasive method may help clinicians identify patients with a more aggressive tumor profile and guide personalized treatment strategies.

## 1. Introduction

Radiogenomics explores the connection between medical imaging features and underlying genetic profiles [1,2]. Through imaging modalities, it is possible to visualize large-scale manifestations of molecular alterations, collectively referred to as radiologic phenotypes [1,2]. The Cancer Genome Atlas (TCGA) Research Network provides an extensive repository of genetic and mutational data specifically related to clear cell renal cell carcinoma (ccRCC) [3,4].

Visual system homeobox1 (VSX1) is a transcription factor that features a paired-like homeodomain and interacts with the locus control region of the visual pigment gene cluster [5]. VSX1 is crucial for the development of the craniofacial structures and eyes, and studies have indicated that mutations in VSX1 contribute to the pathogenesis of posterior polymorphous corneal dystrophy [6,7,8].

It has been demonstrated that VSX1 functions as a potential oncogenic activator in ccRCC, with researchers analyzing the TCGA database to highlight its role in enhancing tumor aggressiveness in vitro. Data derived from the Gene Expression Omnibus and TCGA databases, along with clinical cancer specimens obtained from their department, indicated that VSX1 was upregulated in ccRCC tissues compared to adjacent noncancerous tissues. Furthermore, elevated VSX1 mRNA expression was significantly associated with an advanced T stage, distant metastasis, and a higher pathological stage, establishing it as a crucial prognostic biomarker in ccRCC patients [9].

To date, no studies have investigated the radiogenomic features related to VSX1 expression in patients with ccRCC. The present work aims to examine specific computed tomography (CT) features that may be linked to VSX1 expression in this patient population. Considering the gene’s biological function, it is hypothesized that its expression could be associated with a more aggressive imaging phenotype. This study suggests that CT imaging could serve as a non-invasive tool to gain meaningful insights into the expression patterns of VSX1 in ccRCC.

## 2. Methods

### 2.1. The Cancer Genome Atlas

The TCGA project represents an extensive resource that compiles genomic alterations across more than 20 cancer types, including ccRCC. It was initiated and supported by the National Cancer Institute in partnership with the National Human Genome Research Institute.

Various research institutions provided numerous tissue samples, which underwent thorough genomic profiling using diverse analytical platforms.

The Cancer Imaging Archive is a database that stores anonymized pre-treatment medical imaging data in DICOM format. Funded by the National Cancer Institute, this archive connects imaging data with tissue samples through a unique identifier corresponding to TCGA specimen records. The dataset is openly accessible for public download [10].

### 2.2. Imaging Features

The CT imaging features evaluated for each ccRCC lesion included tumor dimensions (measured in millimeters), margin definition (classified as either well-defined or ill-defined), structural composition (categorized as either solid or cystic), and tumor necrosis (assessed only in solid tumors, with percentages categorized as 0%, 1–33%, 34–66%, or greater than 66%) [11,12,13]. Additional factors included tumor growth pattern (endophytic, less than 50% exophytic, or at least 50% exophytic), presence of calcifications (either present or absent), location in the kidney (left or right), and the existence of venous collateral vessels (dilated renal capsular veins identified through CT or MRI) [11,12,13].

Other key aspects analyzed were involvement of adjacent tissues, invasion of the urinary collecting system, internal tumor hemorrhage, presence of hydronephrosis, and thrombosis in the renal artery or renal vein [11,12,13]. Two further CT indicators were the presence or absence of perirenal fat stranding and thickening of Gerota’s fascia [12,13]. Tumor size was established by measuring the largest axial diameter on postcontrast scans [11,12,13]. A tumor was classified as having well-defined margins if over 90% of its perimeter appeared distinctly demarcated in postcontrast images, including its interface with the renal parenchyma, collecting system, renal sinus, and perinephric fat. The window width and level parameters used for assessment were W: 400 and L: 50 [11,12,13].

Lesions containing at least 50% cystic areas with fluid attenuation values of 20 Hounsfield Units (HU) or lower were labeled as cystic. Conversely, tumors with no cystic portions or those with less than 50% of their volume occupied by cystic spaces were considered solid [11,12,13]. Areas of necrosis were identified as hypodense regions without contrast enhancement, lacking a defined wall, and displaying indistinct margins, distinguishing them from cystic regions [11,12,13]. The extent of necrosis in solid tumors was analyzed during the nephrographic or excretory phases [11,12,13].

Calcifications were identified as high-density deposits or specks. In cases of uncertainty, calcifications were confirmed if the highest HU measurement exceeded 60 HU [11,12,13]. Internal hemorrhage within the tumor was detected by identifying intratumoral regions with HU values matching those of blood (+30 to +80 HU) [11,12,13]. To differentiate between calcifications and hemorrhagic areas with similar HU values, two experienced oncologic radiologists (F.G., with 9 years of experience, and C.A.M., with 13 years of experience) assessed the morphological characteristics of the lesions [11,12,13].

Tumor infiltration was defined as malignant spread into neighboring normal tissues, observed in postcontrast imaging phases [11,12,13]. Hydronephrosis was diagnosed upon identifying urinary tract dilation in postcontrast scans [11,12,13]. Thrombosis in the renal artery or vein was established by detecting filling defects within the vessel lumen on postcontrast images [11,12,13]. Collecting system involvement was confirmed when filling defects were observed in the urinary system during the excretory phase [11,12,13].

### 2.3. Statistical Analyses

Descriptive statistics included frequencies and proportions for categorical variables, and medians with interquartile ranges (IQRs) for continuous variables. The association between VSX1 expression status (positive versus negative) and both clinical–pathological parameters and CT-derived tumor characteristics was assessed. To evaluate statistical differences in medians and proportions between groups, the Wilcoxon rank-sum test, Pearson’s Chi-square test, and Fisher’s exact test were employed, as appropriate. All statistical tests were two-tailed, with significance defined at a *p*-value threshold of less than 0.05. Data analysis and visualization were performed using R statistical software (version 4.1.2, R Foundation for Statistical Computing, Vienna, Austria).

We conducted a penalized logistic regression analysis using the Lasso (Least Absolute Shrinkage and Selection Operator) method to identify the clinicopathological and radiological variables most strongly associated with the expression of the VSX1 gene in ccRCC. The dataset included patients stratified into VSX1-positive and VSX1-negative groups and comprised variables such as sex, tumor necrosis, clinical stage, and advanced imaging features (e.g., collecting system invasion and perinephric fat stranding). Lasso regression enabled automatic variable selection and reduced the risk of overfitting, particularly useful in the context of a relatively small and imbalanced sample.

The Lasso analysis was performed in April 2025 using ChatGPT, an AI model developed by OpenAI (San Francisco, California, United States). The analysis was conducted via the GPT-4 architecture, which includes capabilities for statistical modeling and code generation.

Based on the variables selected by the Lasso model, a composite predictive score for VSX1 gene expression was developed. Each variable was weighted according to its normalized coefficient from the Lasso regression, generating a continuous score for each patient. The model was evaluated using a ROC curve to assess its discriminative ability, and the distribution of scores was compared between VSX1-positive and -negative groups to visualize potential distinguishing patterns.

Following the development of a composite predictive score based on variables selected by the Lasso model, we extended the analysis by integrating a probability estimation component. A simple logistic regression model was trained using the total score as the sole predictor of VSX1 expression. This allowed us to generate a continuous probability curve linking each score value to the likelihood of VSX1 positivity. The curve was then used to construct a complementary probability scale, enhancing the interpretability of the nomogram and allowing for direct clinical translation of the score into estimated genetic risk.

## 3. Results

Data from an overall cohort of 194 patients with available clinical–pathological and CT-based features were extracted. VSX1 gene expression was positive in 37 (19.1%) vs. negative in 157 (80.9%) patients (Table 1).

Patients with a positive VSX1 gene expression were more frequently males (81.1% with VSX1-positive expression vs. 61.1% with VSX1-negative expression, *p* = 0.022). No other clinically meaningful or statistically significant differences emerged between the two patient cohorts (Table 1).

**Figure 1:**
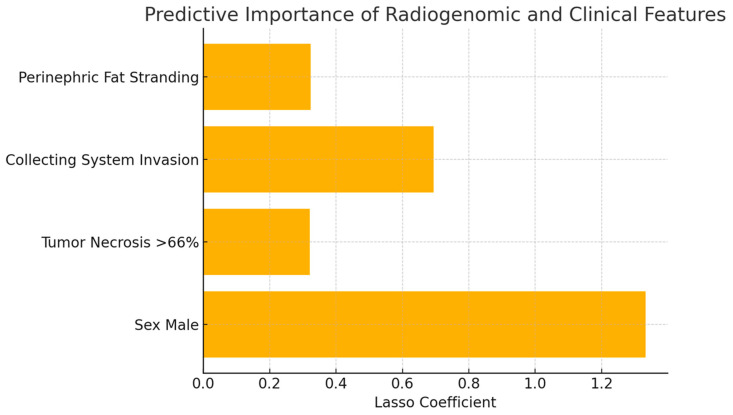
Lasso logistic regression coefficients for variables associated with VSX1 expression in ccRCC. The model identified male sex, tumor necrosis > 66%, collecting system invasion, and perinephric fat stranding as the most relevant predictors. Stage IV was excluded by the model due to lack of additional predictive contribution.

The Lasso logistic regression model identified four informative predictors of VSX1 gene expression: male sex, tumor necrosis > 66%, collecting system invasion, and perinephric fat stranding. Stage IV was excluded, as it did not contribute additional discriminative power to the model. The model demonstrated good discriminative ability, with an area under the curve (AUC) of 0.75 and an overall accuracy of 84.5%. However, sensitivity remained limited, correctly identifying only 7 out of 37 VSX1-positive patients. The coefficient plot highlighted the stronger weight of male sex and collecting system invasion. These findings support the existence of a radiogenomic association between imaging features and the aggressive gene expression profile of VSX1, suggesting potential implications for risk stratification and personalized treatment in ccRCC patients (Figure 1).

**Figure 2:**
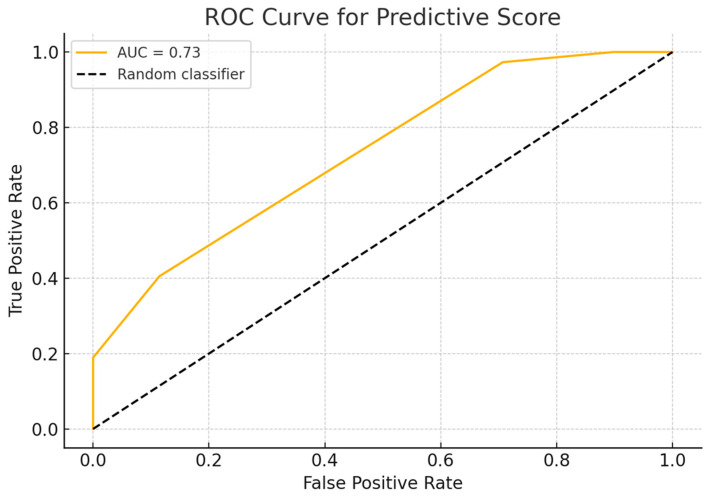
ROC curve of the predictive score for VSX1 expression. The model shows good discriminative performance with an AUC of 0.73, indicating its potential utility in distinguishing VSX1-positive from VSX1-negative cases.

**Figure 3:**
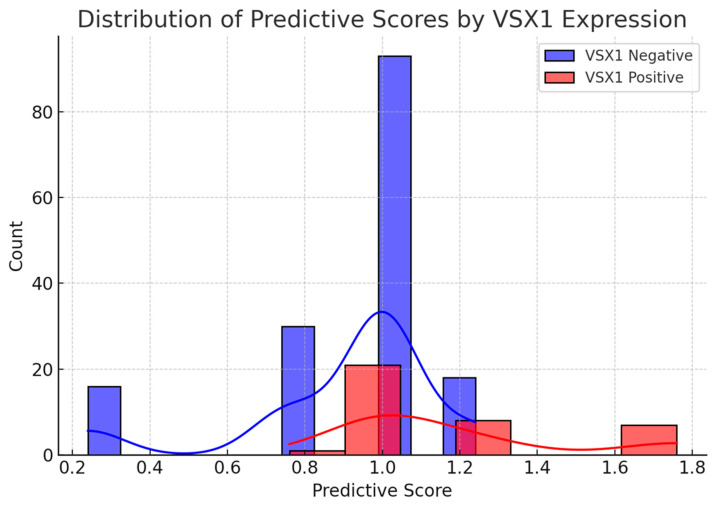
Distribution of predictive scores stratified by VSX1 expression. Patients with positive VSX1 expression tend to have higher scores, although some overlap with VSX1-negative cases is observed, reflecting the probabilistic nature of the model.

The predictive score showed good performance, with an area under the curve (AUC) of 0.73 and an overall accuracy of 79.4% using the median score as a decision threshold. The ROC curve demonstrated a satisfactory separation between the two groups (Figure 2). Additionally, the distribution analysis revealed that VSX1-positive patients tended to have higher scores than -negative ones, with partial overlap (Figure 3). These findings support the score’s utility as a risk stratification tool based on radiological and clinical features associated with a more aggressive tumor phenotype.

**Figure 4:**
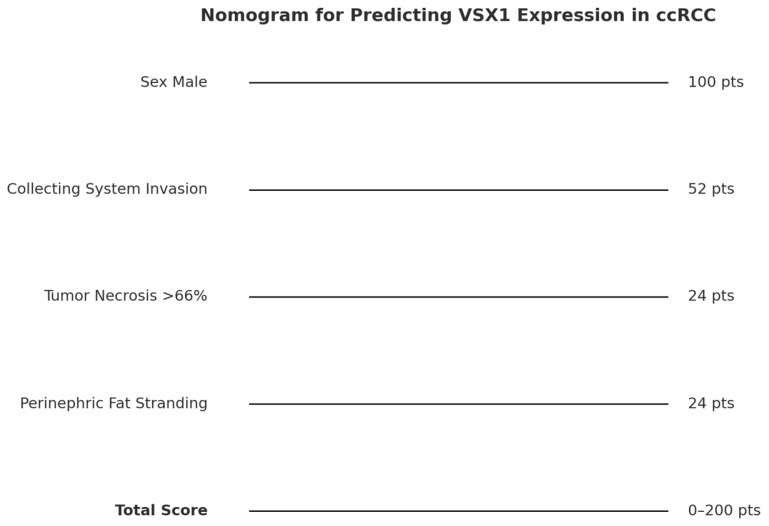
Clinical nomogram for predicting VSX1 expression in ccRCC. Each selected variable contributes a weighted score based on its importance in the Lasso model. The total score can be used to estimate the likelihood of VSX1 positivity.

**Figure 5:**
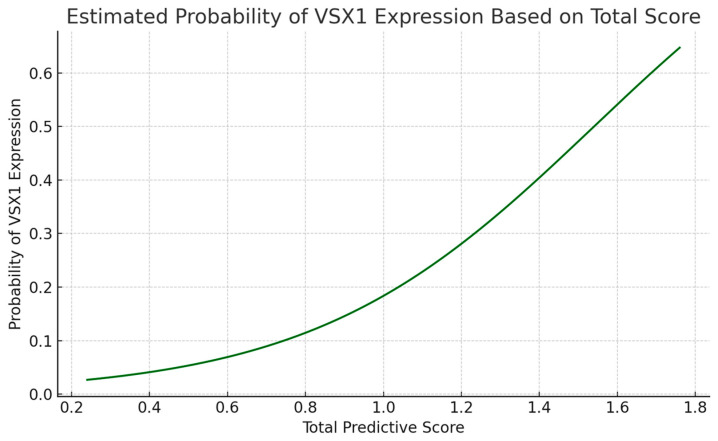
Estimated probability of VSX1 expression as a function of the total predictive score. The curve shows a clear increase in risk associated with higher scores, allowing for probabilistic interpretation of the nomogram output.

The probability curve demonstrated a clear and gradual increase in the likelihood of VSX1 expression as the predictive score rose, confirming the score’s validity as a risk indicator. Patients with higher scores showed a substantially increased estimated probability of harboring a genetically aggressive tumor profile (Figure 4 and Figure 5). This probabilistic interpretation complements the nomogram by providing clinicians with a practical tool to estimate VSX1 expression risk preoperatively. Combined with imaging and clinical data, this approach may support more personalized decision-making in the management of patients with ccRCC.

## 4. Discussion

**Figure 6:**
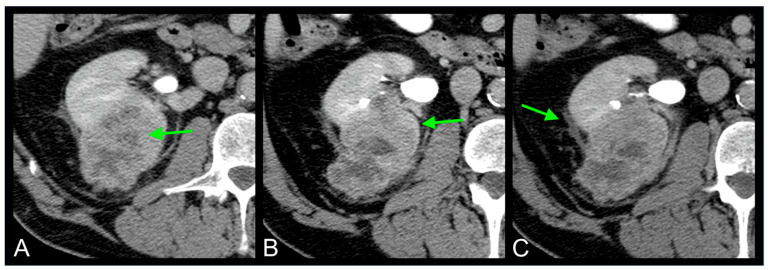
Axial CT images during excretory phase showing radiogenomic features of ccRCC with VSX1-positive expression: extensive tumor necrosis [arrow in (**A**)], collecting system invasion [arrow in (**B**)], and perinephric fat stranding [arrow in (**C**)].

In this study, we explored the clinical and radiogenomic characteristics associated with the expression of VSX1, a gene implicated in tumor aggressiveness, in patients with ccRCC. By comparing patients with positive and negative VSX1 expression, we observed that certain imaging and clinical features, such as male sex, extensive tumor necrosis, collecting system invasion, and perinephric fat stranding, were more frequent in VSX1-positive cases (Figure 6). These observations suggest a potential radiogenomic profile associated with genetically aggressive tumor behavior.

To further investigate these associations, we applied different statistical and machine learning models. Logistic regression with Lasso penalization allowed us to identify the most informative predictors and construct a weighted predictive score. This score showed good performance in discriminating VSX1-positive from VSX1-negative cases, with an AUC of 0.73 and an accuracy of nearly 80%. We then translated this model into a clinical nomogram and complemented it with a probability scale, enabling direct clinical interpretation of the results. These findings support the integration of radiogenomic data into risk stratification tools for ccRCC and highlight VSX1 as a potential biomarker of clinical significance.

The radiogenomic features identified in this study, such as collecting system invasion, extensive tumor necrosis, and perinephric fat stranding, are well-recognized markers of local aggressiveness [14,15,16]. Their strong association with VSX1 expression further supports the hypothesis that this gene is linked to a more aggressive tumor phenotype. The identified radiogenomic features display a specific pattern associated with VSX1 expression in ccRCC.

Renal carcinomas typically exhibit two main forms of necrosis: coagulative necrosis and tumor necrosis [17]. Coagulative necrosis generally results from infarction due to thromboembolic events. It is a widespread, grossly visible form of necrosis and is often linked to a more favorable prognosis. In contrast, tumor necrosis is a microscopic process characterized by the presence of dying or degenerated cells, ghost-like cell remnants, and apoptotic debris. This type of necrosis tends to develop in more aggressive tumors that surpass their vascular supply, resulting in hypoxic conditions and subsequent cell death [17]. Despite ongoing research, the precise mechanisms underlying tumor necrosis remain incompletely understood. It has been demonstrated that an increase in the percentage of necrosis is significantly associated with a higher risk of metastasis and recurrence [17].

Collecting system invasion is a well-known radiological feature of aggressiveness. Anderson et al. reported that the presence of urinary collecting system invasion (UCSI) was independently linked to increased overall and disease-specific mortality in patients who underwent nephrectomy for locally advanced renal cell carcinoma [18]. Similarly, Brookman-Amissah et al. demonstrated that collecting system invasion was a significant independent predictor of reduced cancer-specific survival, and it was frequently associated with synchronous metastatic disease at surgery and multifocal tumor spread [19].

The radiogenomic features associated with disintegrin and metalloproteinase domain-containing protein 12 (ADAM12) expression in ccRCC include primary tumor size, ill-defined margins, tumor necrosis, and collecting system invasion [12]. Notably, the latter two features, tumor necrosis and collecting system invasion, are also observed in ccRCC with VSX1 expression, highlighting a shared radiogenomic profile between the two [12]. However, these gene expressions differ in other aspects: primary tumor size and ill-defined margins are specific to ccRCC with ADAM12 expression, while perinephric fat stranding is uniquely associated with VSX1 expression [12].

Compared to ccRCC with adipose differentiation-related protein (ADFP) expression, ccRCC with VSX1 expression shares the presence of collecting system invasion [13]. However, VSX1 expression is also characterized by extensive tumor necrosis and perinephric fat stranding, which distinguish it from ccRCC with ADFP expression [13]. In summary, VSX1-positive expression in ccRCC has been significantly linked to specific radiological features, including extensive tumor necrosis, collecting system invasion, and perinephric fat stranding. These associations suggest that tumors expressing VSX1 tend to exhibit a more aggressive radiologic profile. Leveraging non-invasive imaging alongside genomic data provides valuable prognostic information, deepens our understanding of underlying gene expression pathways, and opens up new possibilities for the development of tailored therapeutic strategies.

This study has several limitations that should be taken into account. First, its retrospective design and reliance on public datasets such as TCGA and The Cancer Imaging Archive may introduce selection bias and limit the generalizability of the findings. Second, the relatively low number of cases with VSX1-positive expression (19.1%) resulted in an imbalanced dataset, potentially impacting the sensitivity and overall predictive performance of the statistical models, even with the use of Lasso regularization.

Moreover, the CT imaging data were obtained from multiple institutions with differing acquisition protocols, which may have introduced heterogeneity in image quality and interpretation. Although image evaluation was conducted by experienced radiologists using standardized criteria, interobserver variability remains a possible source of bias. Finally, while the predictive score and nomogram demonstrated promising performance, external validation using larger, independent, and prospective cohorts is necessary to confirm their clinical utility and reliability. In line with this, further studies involving larger sample sizes, ideally from a single large institution or a consortium of institutions, will be essential to advance this research and develop it into a clinically meaningful and applicable concept.

Radiogenomics is poised to revolutionize ccRCC management by enabling the non-invasive prediction of genetic mutations and molecular subtypes through imaging features, as demonstrated by models achieving high accuracy in identifying mutations such as von Hippel–Lindau (VHL) and BRCA1-associated protein-1 (BAP1) [20].

However, to transition these advancements into clinical practice, further research is needed to validate robust radiogenomic biomarkers and standardize methodologies across studies.

## 5. Conclusions

This study identified a distinct radiogenomic profile associated with VSX1 expression in ccRCC, characterized by extensive tumor necrosis, collecting system invasion, and perinephric fat stranding features commonly linked to tumor aggressiveness. The predictive model developed using CT imaging and clinical data showed promising performance in estimating VSX1 expression and may serve as a non-invasive tool for risk stratification and personalized treatment planning.

## Figures and Tables

**Table 1:** Descriptive characteristics of the study population stratified according to VSX1 expression.

Characteristic	N	Overall, N = 194 ^1^	VSX1 = 0, N = 157 (81%) ^1^	VSX1 = 1, N = 37 (19%) ^1^	*p*-Value ^2^
**Age**	194	59 (52, 69)	58 (51, 67)	63 (54, 74)	0.077
**Primary tumor size**	194	53 (38, 81)	54 (37, 80)	51 (39, 81)	>0.9
**Sex**	194				0.022
Female		68 (35%)	61 (39%)	7 (19%)	
Male		126 (65%)	96 (61%)	30 (81%)	
**Race**	194				>0.9
White		177 (91%)	143 (91%)	34 (92%)	
Not White		17 (8.8%)	14 (8.9%)	3 (8.1%)	
**Laterality**	194				0.8
Left		92 (47%)	75 (48%)	17 (46%)	
Right		102 (53%)	82 (52%)	20 (54%)	
**Tumor grade** (Fuhrman)	194				>0.9
Grade 1–2		80 (41%)	65 (41%)	15 (41%)	
Grade 3–4		114 (59%)	92 (59%)	22 (59%)	
**Tumor stage** (American Joint Committee on Cancer)	194				>0.9
1		103 (53%)	84 (54%)	19 (51%)	
2		18 (9.3%)	14 (8.9%)	4 (11%)	
3		46 (24%)	38 (24%)	8 (22%)	
4		27 (14%)	21 (13%)	6 (16%)	
**History of other neoplasms**	194				0.7
0		157 (81%)	128 (82%)	29 (78%)	
1		37 (19%)	29 (18%)	8 (22%)	
**Collateral vascular supply**	194				0.7
0		84 (43%)	67 (43%)	17 (46%)	
1		110 (57%)	90 (57%)	20 (54%)	
**Tumor margins**	194				0.7
0		126 (65%)	103 (66%)	23 (62%)	
1		68 (35%)	54 (34%)	14 (38%)	
**Tumor composition**	194				0.7
0		14 (7.2%)	11 (7.0%)	3 (8.1%)	
1		180 (93%)	146 (93%)	34 (92%)	
**Tumor necrosis**	194				0.6
1		11 (5.7%)	9 (5.7%)	2 (5.4%)	
2		114 (59%)	92 (59%)	22 (59%)	
3		48 (25%)	41 (26%)	7 (19%)	
4		21 (11%)	15 (9.6%)	6 (16%)	
**Tumor growth pattern**	194				0.7
0		13 (6.7%)	11 (7.0%)	2 (5.4%)	
1		58 (30%)	49 (31%)	9 (24%)	
2		123 (63%)	97 (62%)	26 (70%)	
**Calcifications**	194				0.7
0		156 (80%)	127 (81%)	29 (78%)	
1		38 (20%)	30 (19%)	8 (22%)	
**Thrombosis or infiltration of renal artery**	194				0.6
0		190 (98%)	154 (98%)	36 (97%)	
1		4 (2.1%)	3 (1.9%)	1 (2.7%)	
**Thrombosis or infiltration of renal vein**	194				>0.9
0		179 (92%)	145 (92%)	34 (92%)	
1		15 (7.7%)	12 (7.6%)	3 (8.1%)	
**Collecting system invasion**	194				0.3
0		135 (70%)	112 (71%)	23 (62%)	
1		59 (30%)	45 (29%)	14 (38%)	
**Intralesional hemorrhage**	194				0.6
0		190 (98%)	154 (98%)	36 (97%)	
1		4 (2.1%)	3 (1.9%)	1 (2.7%)	
**Hydronephrosis**	194				0.6
0		188 (97%)	151 (96%)	37 (100%)	
1		6 (3.1%)	6 (3.8%)	0 (0%)	
**Signs of infiltrations**	194				>0.9
0		189 (97%)	153 (97%)	36 (97%)	
1		5 (2.6%)	4 (2.5%)	1 (2.7%)	
**Perinephric fat stranding**	194				0.3
0		93 (48%)	78 (50%)	15 (41%)	
1		101 (52%)	79 (50%)	22 (59%)	
**Gerota’s fascia thickening**	194				0.5
0		120 (62%)	99 (63%)	21 (57%)	
1		74 (38%)	58 (37%)	16 (43%)	

^1^ Median (IQR); n (%). ^2^ Wilcoxon rank-sum test; Pearson’s Chi-square test; Fisher’s exact test.

## Data Availability

The original data presented in the study are openly available in [The Cancer Genome Atlas Kidney Renal Clear Cell Carcinoma Collection (TCGA-KIRC) (Version 3) [Data set]] [https://doi.org/10.7937/K9/TCIA.2016.V6PBVTDR] [4].
